# Liraglutide improves antioxidant defense in hearts of spontaneously hypertensive female rats independently of changes in blood pressure in a pre-clinical model of menopause

**DOI:** 10.1590/1414-431X2025e14209

**Published:** 2025-04-14

**Authors:** W.C. dos Santos, S.N. Ronchi, L.A. Gonçalves, L.C.S.L. Oliveira, G.J. Sousa, A.F. Melo, T.U. de Andrade, N.S. Bissoli, G.A. Brasil

**Affiliations:** 1 Departamento de Ciências Farmacêuticas, Universidade de Vila Velha, Vila Velha, ES, Brasil; 2 Departamento de Ciências Fisiológicas, Universidade Federal de Espírito Santo, Vitória, ES, Brasil

**Keywords:** GLP-1, Estrogen, Menopause, Antioxidant, Hypertension

## Abstract

Liraglutide (LIRA) is an agonist of the GLP-1 receptor used in the treatment of type 2 diabetes with a cardioprotective effect, although little is known about the effects of LIRA in post-menopause. We aimed to evaluate the effects of LIRA in the cardiovascular system of ovariectomized spontaneously hypertensive rats (SHR). SHR rats were separated into two groups: ovariectomized (saline) and ovariectomized + liraglutide (0.6 and 1.2 mg/kg for 4+4 weeks, respectively). Systolic blood pressure (SBP) was indirectly evaluated at the beginning and end of treatment. Diastolic, systolic, and mean blood pressure were evaluated in the carotid artery of anesthetized animals, while left ventricle systolic blood pressure (LVSBP) and left ventricle derivatives (-dP/dt; +dP/dt) were evaluated in the left ventricle. An oral glucose tolerance test (GTT) was conducted. Antioxidant enzymes and calcium-handling proteins were analyzed in heart tissue by western blot. Treatment with LIRA increased the expression of antioxidant enzymes (superoxide dismutase (SOD2) and catalase). No changes were observed in the GTT, cardiac hemodynamics, blood pressure, and calcium-handling protein expression. A decrease in visceral fat depot was observed without changes in final body weight. LIRA induced an antioxidant subclinical effect in ovariectomized SHR female rats without changing glucose metabolism and cardiac blood pressure.

## Introduction

Liraglutide (LIRA) is a therapeutic option for the treatment of glycemic alterations and/or type 2 diabetes mellitus (DM2) (1,2), acting as an agonist of glucagon-like peptide 2 (GLP-1) receptors (1). GLP-1 is a peptide from the incretin family, which includes gut hormones, that promotes insulin release without causing hypoglycemia (3).

In addition to the pancreas, GLP-1 receptors (GLP1-R) are also expressed in the gastrointestinal tract, heart, lung, kidney, and brain (4). In extrapancreatic tissues, one of the roles played by GLP-1 is vascular tonus modulation, as its receptors are found in coronary endothelial cells and smooth muscle cells, where it promotes nitric oxide (NO) production and vasodilatation (5).

In the heart, GLP-1 has been shown to improve cardiac performance, both in animal models (6) and in humans (7) after acute myocardial infarction. In addition, another study showed that LIRA has anti-inflammatory and antioxidant effects in cardiomyocyte cultures, suggesting that it has cardioprotective activity (8).

Cardiovascular disease (CVD) is the leading cause of death in the world and is more common in men than in women (9), although the numbers match after menopause (10). The protection against CVD in women is associated with higher levels of endogenous estrogen (E2), which decreases abruptly after menopause.

Moreover, the increased incidence of CVD in post-menopausal women is associated to the increased oxidative stress at this stage (11). Oxidative stress is caused by an increased production of reactive oxygen species (ROS) or by a decrease in the endogenous antioxidant system, formed by proteins and enzymes such as superoxide dismutase (SOD) and catalase. It has been shown that the decrease in estrogen leads to an increase in oxidative stress (12), which could be associated with worsening of vascular response and further damage to heart and kidney tissues (13).

Estrogen has several beneficial effects on the cardiovascular system (CVS), such as by enhancing the production of NO and promoting anti-inflammatory and antioxidant activities (14-16). Nonetheless, many studies have reported that estrogen replacement during menopause is controversial with regards to CVD (17). Thus, the search for new therapeutic alternatives to prevent and/or treat CVD in perimenopause women is a pressing one.

Despite the knowledge about the effects of LIRA on CVD, little is known about its effects on post-menopausal women. Our aim here was to evaluate the effects of chronic treatment with LIRA on hemodynamic parameters and in the expression of antioxidant and calcium-handling proteins in a pre-clinical model of menopause and hypertension in ovariectomized spontaneously hypertensive rats (SHR).

## Material and Methods

### Animals

The study was conducted with female SHR (2 months of age) weighing 150 g. The animals were provided by the Laboratório de Acompanhamento Experimental do Complexo Biopráticas of the Vila Velha University (UVV) and were kept in collective cages (5 animals/cage) with free access to water and food (standard chow - Moinho Primor^®^, Brazil), a 12-h light/dark cycle, and controlled temperature and humidity. All procedures were performed as recommended by the Guide for the Care and Use of Laboratory Animals of the National Institutes of Health (18) and previously approved by the Ethics Committee for Animal Welfare of the UVV (Opinion No. 540-2019).

### Ovariectomy

Ovariectomy was performed 24 h before the start of treatment under anesthesia with ketamine (80 mg/kg, intraperitoneal (*ip*), Syntec do Brasil Ltda., König Laboratories, SA, Brazil) and xylazine (12 mg/kg, *ip*, König Laboratories, SA). Bilateral dorsolateral incisions were made in the skin and the underlying muscle tissue was dissected to locate the ovaries and fallopian tubes. The ovaries were then removed, and the muscle and skin sutured with absorbable suture.

### Experimental protocol

The animals were randomly separated into two groups: ovariectomized control group (O), in which the animals were submitted to ovariectomy and treated with vehicle by subcutaneous injection (*sc*) of saline solution (0.9% NaCl), in a volume similar to that received by the group treated with LIRA and ovariectomized group treated with LIRA (OL), in which the ovariectomized animals received liraglutide (Novo Nordisk Pharma Ltd., Denmark) in the following treatment schedule: 0.6 mg/kg once daily in the first four weeks, followed by 0.6 mg/kg twice daily for another four weeks.

The animals were weighed at baseline, weekly for dose adjustment, and at the end of treatment. At the end of the treatment, the animals were submitted to the glucose tolerance test and hemodynamic evaluation.

### Fasting blood glucose and glucose tolerance test

The glucose tolerance test (GTT) was performed as previously described (19). Briefly, after fasting for 12 h, glucose (2 g/kg, *ip*) was administered and blood glucose was measured in tail blood samples after 0, 5, 10, 20, 30, 60, and 120 min using a glucometer (OnCall Plus II, Acon^®^, USA).

### Catheterization and hemodynamic measurements

After the experimental protocol, the animals were anesthetized with ketamine and xylazine (100 and 10 mg/kg *ip*, Syntec do Brasil Ltda; König Laboratories, SA) for hemodynamic measurements. All measurements were taken with the animals placed on a warm table to maintain their temperature. A polyethylene catheter (PE-50) filled with saline (0.9% NaCl) was inserted into the right carotid artery. The catheter was connected to a pressure transducer (Biopac System^®^, MP100-CE, USA) coupled to a biological data acquisition system (Biopac Systems^®^). Systolic blood pressure (SBP), diastolic blood pressure (DBP), and mean blood pressure (MBP) data were recorded for fifteen minutes in the carotid artery. The catheter was then guided to the left ventricle (LV) for the acquisition of the left ventricular end-systolic pressure (LVESP), the maximum rate of ventricular pressure increase or the peak positive value of the first derivative of the left ventricular pressure (+dP/dt, mmHg/s), the rate of pressure decay (−dP/dt, mmHg/s), and the time constant of isovolumic relaxation of the LV (Tau, s). The signal is expressed in mmHg/s. After the procedure, the catheter was removed from the LV, and DBP was measured again. Animals that showed a reduction in DBP greater than 10 mmHg would be excluded from the study, as that indicates damage to the aortic valve, however no animal needed to be excluded from the present study. Data were analyzed using LabChart software, version 7 (AD Instruments, Australia).

### Ponderal data

After hemodynamic evaluation, the animals were euthanized by exsanguination while still anesthetized. The organs were removed (LV, uterus, and perirenal and inguinal fat), cleaned in phosphate buffer (PBS, pH=7.2), dried, and weighed. The tibia was removed, cleaned, and measured. The ratio of organ weight to tibial length (g/mm) was used as a parameter for organ hypertrophy. The LV was stored at -80°C for the assessment of protein expression.

### Western blotting

LV samples (100 mg) were homogenized in lysis buffer [Tris-HCL pH 7.4 (10 mM), PMSF (1 mM), NaVO_3_ (1 mM), SDS (1%), DTT (0.5 mM), EDTA (5 mM), and protease inhibitor cocktail (1:100)] and the total protein content was measured using the Bradford assay. Proteins (100-mg samples) were separated by electrophoresis using 7.5-10% SDS PAGE. Nitrocellulose membranes were blocked with 5% non-fat milk and exposed to the following primary antibodies: antiSERCA2a (1:1000, LOT UI2839274, Affinity BioReagents, USA); anti-PLB (1:1000, LOT TI270655, Invitrogen, USA); anti-p-Ser16-PLB (1:1000, LOT RK243708, Invitrogen); anti-SOD 1 (1:200, LOT 0801 Sigma, USA); anti-SOD 2 (1:1000, LOT 23356, BD Laboratories, USA); catalase (CAT) (1:2000, LOT E1418, Sigma); and anti-glyceraldehyde-3-phosphate dehydrogenase (GAPDH) (1:4000, LOT 015M4824V, Merck Millipore, Germany). The membranes were then incubated with the secondary antibodies (anti-mouse monoclonal IgG), which were peroxidase-conjugated (1:5000, Abcam, USA). Bands were quantified using the Bio-Rad Image Lab 5.2.1 software and protein expression levels were normalized using GAPDH.

### Statistical analysis

Statistical analysis was performed using the GraphPad Prism software, version 8 (GraphPad Software, USA). Data are reported as means±SE and normality was assessed by the Kolmogorov-Smirnov method. Data were compared using unpaired Student's *t*-test. Data in which the time had an influence on the evaluated parameter was analyzed by two-way analysis of variance (ANOVA).

## Results

### Ponderal data

Ovariectomy led to equal weight gain in both treatments. LIRA treatment had no impact on the uterus and the LV/TB ratio (Table 1). In addition, LIRA induced a decrease in fat depot ratio (g/mm), without altering LV/TB and U/TB.

**Table 1:** Ponderal data of ovariectomized animals submitted (OL) or not (O) to liraglutide treatment.

Parameters	Groups		
	O	OL	P value
Initial body weight (g)	151.6±16.6	149.0±3.6	0.8954
Final body weight (g)	209.2±8.07	197.1±5.77	0.2437
LV/TB (g/mm)	0.01923±0.0007	0.02012±0.001	0.3242
U/TB (g/mm)	0.00186±0.0001	0.002062±0.0002	0.4822
PRF/TB (g/mm)	0.01886±0.001	0.004653±0.0008*	<0.0001*
IF/TB (g/mm)	0.06125±0.008	0.03332±0.0059*	0.0209*

LV: Left ventricle; U: uterus; PRF: perirenal fat; IF: inguinal fat; TB: tibial length. Data are reported as means and SE (n=7 per group). *P<0.05, Student's *t*-test.

### Fasting blood glucose and GTT

**Figure 1:**
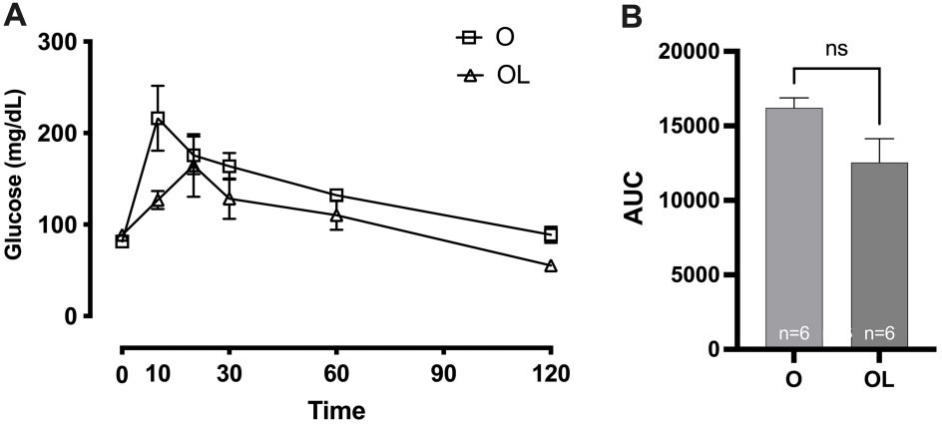
Glucose oral tolerance test results over time (h). **A**, Representative curves of the ovariectomized control group (O) and ovariectomized group treated with liraglutide (OL). **B**, Area under the curve (AUC). Data are reported as means±SE. The difference between groups was evaluated by Student's *t*-test. The data presented a parametric distribution. ns: non-significant.

No differences were observed between groups regarding glucose levels (Figure 1A and B; O: 16208±668.8, OL: 12541±1593 AUC), although there was a tendency to a decrease in the area under the curve (P=0.0598) in the group treated with LIRA.

### Hemodynamic parameters

After 60 days of treatment, LIRA did not produce changes in the MBP of animals under anesthesia and no hemodynamic parameter was changed (Table 2).

**Table 2:** Hemodynamic data from ovariectomized animals submitted (OL) or not (O) to treatment with liraglutide for 60 days.

Parameters	Groups		
	O	OL	P value
MBP (mmHg)	113.2±5.7	105.6±3.9	0.2901
SBP (mmHg)	134.5±4.6	127.0±4.3	0.3093
DBP (mmHg)	94.9±5.7	85.9±4.0	0.2143
HR (bpm)	208.5±6.2	185.0±15.3	0.1751
dP/dt max (mmHg/s)	2916±140.8	2656±139.2	0.2366
dP/dt min (mmHg/s)	-2778±204.7	-2163±186.1	0.0619
TAU (s)	0.041±0.001	0.049±0.003	0.1174

MPB: mean blood pressure; SBP: systolic blood pressure; DBP: diastolic blood pressure; HR: heart rate; dP/dt: left ventricle derivatives; TAU: time constant of isovolumic relaxation. Data are reported as means±SE (n=7 pre group). P<0.05, Student's *t*-test.

### Western blot

**Figure 2:**
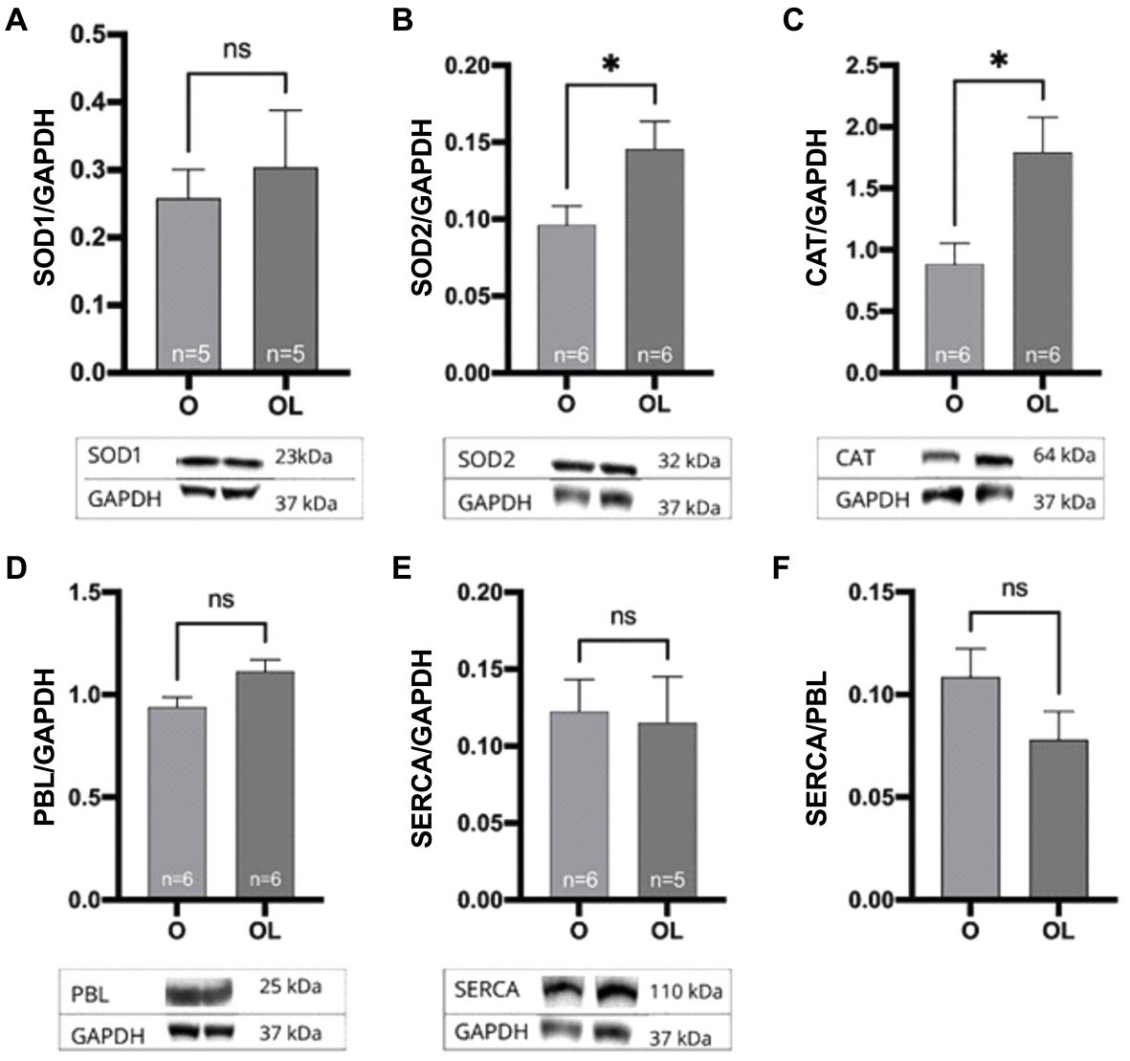
Relative protein expression in hearts of ovariectomized rats treated (OL) or not (O) with liraglutide. **A**, Superoxide dismutase 1 (SOD1; P=0.61); **B**, superoxide dismutase 2 (SOD2; P=0.04); **C**, catalase (CAT; P=0.02); **D**, phospholambam (PBL; P=0.05); **E**, SERCA (P=0.84); **F**, SERCA/PBL ratio (P=0.0858). The expression of each protein was normalized by GAPDH. Data are reported as means±SE. *P<0.05, Student's *t*-test; ns: non-significant.

The evaluation of antioxidant enzyme expression revealed an increase in SOD2 (Figure 2B; O: 0.09613±0.01249; OL: 0.1455±0.01811 AU) and CAT (Figure 2C; O: 0.8852±0.1697; OL: 1.793±0.2845 AU) in the OL group. On the other hand, no differences were observed in SOD1 (Figure 2A) and calcium-handling protein expressions (PBL, SERCA, and PBL/SERCA ratio; Figure 2D-F).

## Discussion

Our study showed, for the first time, that 60 days of treatment with LIRA in female SHR increased the expression of antioxidant proteins in heart tissue without promoting changes in cardiac hemodynamics and independently of glucose metabolism and hypertension.

Some studies indicate that estrogen has a cardioprotective effect, decreasing the risk of hypertension and cardiovascular events (such as myocardial infarction, stroke, and thromboembolic diseases) in pre-menopause women compared with men of the same age (20). In post-menopause, estrogen levels are reduced, and this reduction is associated with an increased incidence of hypertension in women (9). However, estrogen replacement is controversial, especially because of the side effects related to the cardiovascular system (17).

The increase in CVDs observed in post-menopausal women is associated with the redox imbalance that occurs in this population (11), showing that estrogen has an antioxidant action. Thus, the decrease in estrogen levels in menopause leads to a higher production of ROSs, which promotes a pro-oxidant environment in the female body (13,14).

ROS scavenging takes place through an endogenous antioxidant system, which includes enzymes such as SOD, which dismutates the superoxide anion to hydrogen peroxide (H_2_O_2_). Complementarily, the enzyme catalase converts H_2_O_2_ into water, which is inert to the body. The accumulation of ROS increases the risk of developing chronic diseases and is related to the genesis of hypertension (21).

We have also observed that LIRA promoted subclinical antioxidant activity. Ovariectomy has been shown to promote a decrease in the expression of the antioxidant enzymes SOD2 and catalase (12), which is reversed by estrogen supplementation. Thus, we can infer that, in the present study, there was a reduction in the expression of these enzymes caused by estrogen decline in ovariectomized rats, and LIRA was able to improve the antioxidant environment in the heart and prevent heart damage.

Many studies show the effects of LIRA on blood pressure in both humans and animal models of hypertension. Treatment with LIRA (0.1 mg/kg per day) for 8 weeks showed that it does not affect SBP, as well as body weight and glucose in obese Zucker (fa/fa) rats (22). In another study (23), using a model of polycystic ovary syndrome caused by a dihydrotestosterone implant, it was reported that LIRA decreased body weight and mean arterial pressure in these conditions. In addition, a study conducted with male SHR using LIRA (300 µg, twice a day, for 7 weeks) reported that the treatment attenuated hypertension (24). In humans, the effect of LIRA in decreasing blood pressure seems to be modest, as concluded by Zhao et al. (25) in a recent meta-analysis. They observed that LIRA (3.0 mg/kg) significantly decreased DBP by 1.46 mmHg, although a slight increase in DBP (0.47 mmHg) was detected at 1.8 mg/kg compared with placebo (25).

These studies indicate that the effect of LIRA can be influenced by sex, health status, and dose. On the other hand, the cardiac protective effect is independent of blood pressure, as we showed in the present study. Among the aforementioned determinants, sex seems to be important for some effects: it has been shown that, in women, estrogen determines the magnitude of the effect of GLP-1 agonists (26-28). Thus, we can speculate that both estrogen and LIRA act through common intracellular pathways, activating the same kinase proteins to promote their effects (29,30).

In addition to indirect blood pressure evaluation, we catheterized the left ventricle to evaluate whether LIRA could change hemodynamic parameters. In addition to not affecting blood pressure, no changes in hemodynamic parameters or in calcium-handling proteins were observed in OVX animals treated with LIRA. Previous data from our laboratory using SHR (31), as well as studies with ovariectomized Wistar rats (32) and post-menopausal women (33), have shown that estrogen deficiency leads to a reduction in cardiac contractility, even after just eight weeks, which could progress to heart failure over time. Therefore, we refute our hypothesis that LIRA could improve these parameters. Nevertheless, the increase in heart antioxidant defense and the fact that there was no worsening in contractility are positive points to hypertensive females with an estrogen deficiency who use GLP-1 agonists.

In this study, we did not observe body weight loss, although the animals had a decrease in inguinal and perirenal fat depots. Treatment with LIRA was also evaluated in ovariectomized Wistar rats, and prevention of body weight gain and a decrease in body fat depots in treated animals was observed (34).

The distribution of body fat in women changes over time, and post-menopause is characterized by an increase in visceral fat (35). Visceral fat is associated with an increase in vascular resistance, intrarenal pressure, insulin resistance, and pro-inflammatory imbalance (36). LIRA has been shown to effectively reduce both subcutaneous and visceral fat depots, independent of its effects in decreasing glycemia (37).

Therefore, our data showed that LIRA played an important role in decreasing visceral fat depot and increasing antioxidant enzymes in heart tissue in a model of hypertension and post-menopause, which could be associated with possible cardiac and metabolic benefits.

Our study had some limitations. The method used in our protocol was not the gold standard for hemodynamic evaluations, which could produce results not as precise as those obtained with other protocols.
